# Renal Protective Effects of Resveratrol

**DOI:** 10.1155/2013/568093

**Published:** 2013-11-28

**Authors:** Munehiro Kitada, Daisuke Koya

**Affiliations:** Diabetology and Endocrinology, Kanazawa Medical University, 1-1 Daigaku, Uchinada, Kahoku, Ishikawa 920-0293, Japan

## Abstract

Resveratrol (3,5,4′-trihydroxystilbene), a natural polyphenolic compound found in grapes and red wine, is reported to have beneficial effects on cardiovascular diseases, including renal diseases. These beneficial effects are thought to be due to this compound's antioxidative properties: resveratrol is known to be a robust scavenger of reactive oxygen species (ROS). In addition to scavenging ROS, resveratrol may have numerous protective effects against age-related disorders, including renal diseases, through the activation of SIRT1. SIRT1, an NAD^+^-dependent deacetylase, was identified as one of the molecules through which calorie restriction extends the lifespan or delays age-related diseases, and this protein may regulate multiple cellular functions, including apoptosis, mitochondrial biogenesis, inflammation, glucose/lipid metabolism, autophagy, and adaptations to cellular stress, through the deacetylation of target proteins. Previous reports have shown that resveratrol can ameliorate several types of renal injury, such as diabetic nephropathy, drug-induced injury, aldosterone-induced injury, ischemia-reperfusion injury, sepsis-related injury, and unilateral ureteral obstruction, in animal models through its antioxidant effect or SIRT1 activation. Therefore, resveratrol may be a useful supplemental treatment for preventing renal injury.

## 1. Introduction

Chronic kidney disease (CKD),which is characterized by achronic reduction in the glomerular filtration rate (GFR) and the presence of proteinuria or albuminuria, is recognized as an independent risk factor for both end-stage renal disease (ESRD) and cardiovascular disease, leading to a decrease in quality of life and an increased risk of mortality[1]. Acute kidney injury (AKI) is common in the setting of critical illness and is associated with a high risk of death[2]. In addition, AKI can directly cause ESRD and can increase the risks of the development of incident CKD and the worsening of underlying CKD[3]. Therefore,additional treatment to preventboth chronic and acute kidney injury is necessary.

**Figure 1:**
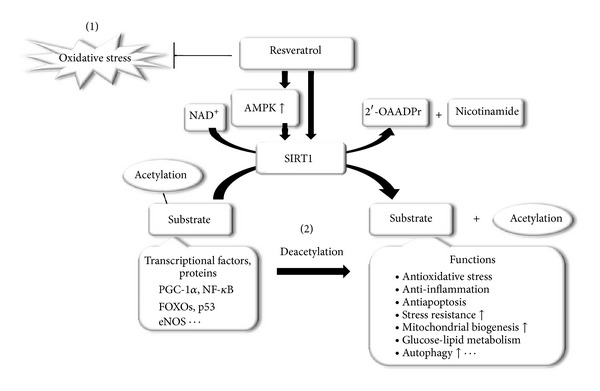
Proposed mechanisms by which resveratrol exerts cytoprotection. (1) Resveratrol attenuates oxidative stress. (2) Resveratrol activates SIRT1, which is an NAD^+^-dependent deacetylase,directly or indirectly via AMPK activation. SIRT1 plays an important role in the regulation of oxidative stress, inflammation, apoptosis, stress resistance, mitochondrial biogenesis, autophagy, and glucose-lipid metabolism, via the deacetylation of many substrates.

Resveratrol (3,5,4′-trihydroxystilbene) is a polyphenolicphytoalexin that occurs naturally in many plant parts and products, such as grapes, berries, red wine, and peanut skins [4], and hasnumerous beneficial health effects. Previous epidemiological studies have revealed an inverse correlation between red wine consumption and the incidence of cardiovascular disease, a phenomenon known as the “French Paradox.” The French populationhas relatively low rates of cardiovascular disease despite traditionally eating a diet rich in saturated fat [5]. Resveratrol,which is presentin red wine,has been postulated to explain the protective effects on the cardiovascular systemobserved in the French Paradox, and the effectsof this compound are exerted through several mechanisms, includingantioxidanteffects[6]. SIRT1, an NAD^+^-dependent deacetylase, has been identified as one of the molecules through which calorie restriction (CR) extends the lifespan and delays age-related diseases [7–9]. The activation of SIRT1 exertscytoprotective effects through multiple mechanisms, such asantiapoptosis, antioxidative, and anti-inflammation effects and the regulation of mitochondrial biogenesis, autophagy, and metabolism in response to the cellular energy and redox status[10].Resveratrol has been shown to be aSIRT1 activator [11], andnumerous previous studieshave shown that the administration of resveratrol can prevent many diseases, such as diabetes, neurodegenerative disorders, cognitive disorders, cancer, kidney diseases, and cardiovascular disease through SIRT1 activation [9, 10, 12]. Thus, resveratrol exerts itscytoprotective effects through at least two mechanisms, antioxidant activity and SIRT1 activation (Figure 1). In the present review, we summarizethe protective effects of resveratrolagainst several types of renal injury and discuss the mechanisms involved.

## 2. Mechanisms of the Cytoprotective Effects of Resveratrol

### 2.1. Resveratrol as an Antioxidant

An excess of reactive oxygen species (ROS) is involved in a variety of diseases, the aging process, and numerous cellular response pathways[13, 14]. ROS include superoxide(O_2_^−^), the hydroxyl radical (OH^•^),and peroxynitrite(ONOO^−^), and these compounds attackcellular proteins and DNA.Oxidative stress is induced by an imbalance between ROS production and antioxidant defenses; therefore, exogenous antioxidants or the modulation of antioxidant enzymes can be expected toreduce oxidative stress.Resveratrol is anatural antioxidant. Previous studieshave shown that resveratrol can directly scavenge ROS, such as O_2_^−^, OH^•^,and ONOO^−^[15, 16]. In addition to scavenging ROS, exogenouslyadministered resveratrolmodulatesthe expression and activity of antioxidant enzymes, such assuperoxide dismutase, glutathione peroxidase (GPx), and catalase, through transcriptional regulation via nuclear factor E2-related factor 2 (Nrf2), activator proteins (AP)-1, forkhead box O (FOXO), and SP-1 or through enzymatic modification[17–20].

### 2.2. Resveratrol Activates SIRT1

Aging is a universal process that affects all organs, and age-related disruptions in cellular homeostasis result in a reduction in responsiveness to physiological stress and organ dysfunction. Numerous studies have revealed that CR retards aging or extends the lifespans of yeast, worms, flies, and rodents[7]. Colman et al. also reported that CR delayed the onset of age-associated pathologies, including diabetes, cancer, cardiovascular disease, and brain atrophy and decreased mortality in rhesus monkeys [21]. In addition, Fontana et al. showed that CR for an average of 6 years improved metabolism in humans, as measured by the levels of serum insulin, cholesterol, C-reactive protein (CRP), and tumor necrosis factor (TNF)-*α* as well as the thickness of the carotid intima media [22]. This group also observed that long-term CR ameliorated declines in left ventricular diastolic function and decreased the levels of serum tumor growth factor (TGF)-*β*1, TNF-*α*, and high-sensitivity CRP [23]. Thus, CR induces these antiaging effects by improving insulin sensitivity and reducing inflammation and oxidative stress, and CR is accepted as the only established experimental antiaging paradigm.

Based on initial studies on aging in yeast, silent information regulator 2 (Sir2), an NAD^+^-dependent deacetylase, was identified as one of the molecules through which CR extends the lifespan and delays age-related diseases [24]. Homologues of Sir2 in higher eukaryotic organisms are known as sirtuins. SIRT1, the sirtuin most closely related to Sir2, is one of seven sirtuins in mammals [9]. The beneficial effects of CR involve the function of SIRT1, which is induced by CR in various tissues [21]. The importance of SIRT1 in the effects of CR has been demonstrated using genetically altered mice[25, 26]. SIRT1 is an important regulator of a wide variety of cellular processes, including stress responses, cell survival, mitochondrial biogenesis, and metabolism in response to the cellular energy, as well as the redox status, via the deacetylation of many substrates[9, 12]. Therefore, SIRT1 activators are expected to function as CR mimetics, andthe screening of compounds for their ability to activate SIRT1 led to the discovery of 18 small molecules, including resveratrol[11]. Resveratrol can activate SIRT1 throughmultiple mechanisms. Although resveratrol was originally thought to directly activate SIRT1 through an allosteric effect, AMPK is required for the activation of SIRT1 by resveratrol. AMPK plays an important role in the regulation of metabolism in response to the energy balance[27]. In addition, Park et al. found that resveratrol activates SIRT1 through the activation of AMPK via the inhibition of phosphodiesterase 4 (PDE 4) and the elevation of cAMP in cells, thereby providing a new mechanism to explain SIRT1 activation by resveratrol[28]. A recent study reported by Price et al. also demonstrated a direct link between SIRT1 and the metabolic benefits of resveratrol[29]. These authors reported that a moderate dose of resveratrol (25–30 mg/kg/day to mice treated with high fat diet) first activated SIRT1 and then induced the deacetylation of liver kinase B (LKB) 1 and the activation of AMPK, leading to increased mitochondrial biogenesis and function. In addition, a high dose of resveratrol (215–235 mg/kg/day to mice treated with high fat diet) may directly activate AMPK, independently of SIRT1. Moreover, Hubbard et al. demonstrated that sirtuin-activating compounds (STACs), including resveratrol, can increase the catalytic activity of SIRT1 toward certain substrates through an allosteric mechanism involving an amino terminal domain near the catalytic core and through direct binding to SIRT1 [30].

## 3. Renal Protective Effects of Resveratrol

### 3.1. Diabetic Nephropathy

Diabetic nephropathy is one of the more serious complications of diabetes and is the most common cause of ESRD. Oxidative stress has been implicated in the pathogenesis of diabetic vascular complications, including nephropathy[31]. Previous studieshave clearly demonstrated that resveratrol can improve diabetic nephropathy in several animal models of types 1 and 2 diabetes through its antioxidative effectsresulting from direct radical scavenging or the modulation of antioxidant enzymes.

Sharma et al. reported that treatment with resveratrol (5 mg or 10 mg/kg orally) for 2 weeks improved urinary protein excretion, renal dysfunction, and renal oxidative stress in streptozotocin- (STZ-) induced diabetic rats [32]. In addition, Palsamy and Subramanian reported that resveratrol treatment (5 mg/kgorally for30days) resultedin significant normalization of the creatinine clearance and the levels of plasma adiponectin, C-peptide, and renal oxidative stress and inflammation in STZ-nicotinamide-induced diabetic rats [33]. Furthermore, resveratrol treatment amelioratedthe dysfunction of antioxidant enzymes, including superoxide dismutase (SOD), catalase, glutathione peroxidase (GPx), glutathione-S-transferase(GST), and glutathione reductase (GR), and the reduction in the levels of vitamin C, vitamin E and reduced glutathione (GSH) in diabetic kidneys. In addition, they found that the expression levels of Nrf2 and its downstream enzyme, including*γ*-glutamyl cysteine synthetase (GCS), m-GST, and hemoxygenase-1 (HO-1) were significantly decreased in the renal tissues of diabetic rats. However, the administration of resveratrol modulates the expression of Nrf2 in the context of diabetes-induced oxidative stress by upregulating *γ*-GCS, m-GST, and HO-1. We also reported thatresveratrol treatment (400 mg/kg, orally, administered at concentration of 0.3% resveratrol) alleviated albuminuria andhistological mesangial expansion andreduced the increased levels of renal oxidative stress and inflammation in the kidneys of db/db mice through the scavenging of ROS and normalizing manganese (Mn)-SOD function by decreasing its levels of nitrosative modification [20]. Kim et al. demonstrated that resveratrol prevents diabetic nephropathy in db/db mice by the phosphorylation of AMPK and SIRT1-peroxisome proliferator-activated receptor *γ* coactivator (PGC)-1*α* signaling, which appear to prevent lipotoxicity-related mesangial cell apoptosis and oxidative stress in the kidney [34]. Zhang et al. showed that high glucose levels enhance mesangial cell proliferation and fibronectin expression through the c-Jun N-terminal kinase (JNK)/nuclear factor *κ*B (NF-*κ*B)/NADPH oxidase/ROS pathway, which was inhibited by resveratrol in cultured mesangial cells [35].

In addition to antioxidant effects, resveratrol has other propertiesthat can ameliorate diabetes or high glucose-induced kidneyinjurybyactivating AMPK or SIRT1. Ding et al. reported that resveratrol treatment attenuates renal hypertrophy and urinary albumin excretion in the early stage of diabetes in STZ-induced diabetic rats without affecting the blood glucose levels [36]. They found that resveratrol activates AMPK and inhibitsthe phosphorylation of 4E-BP1 and S6 in diabetic kidneys. Moreover, in cultured mesangial cells, resveratrol has been shown to blockthe high glucose-induced dephosphorylation of AMPK and the phosphorylation of 4E-BP1 and S6, inhibiting both the DNA synthesis and proliferation. In addition, Lee et al. reported that resveratrol ameliorates high glucose-induced protein synthesis in glomerular epithelial cells [37]. Resveratrol increases AMPK phosphorylation and abolishes high glucose-induced reductions in the AMPK phosphorylation level. In addition, resveratrolinhibitsthe high glucose-induced phosphorylation of eIF4E, eEF2, eEF2 kinase, and p70S6 kinase, which have significant roles in the initiation and elongation steps of mRNA translation. Resveratrol prevents the high glucose-induced hyperacetylation of LKB-1, which is an upstream regulator of AMPK, leading to AMPK activation, and the deacetylation of LKB-1 isindependent of SIRT1. However, Tikoo et al. showed that resveratrol (55 mg/kg, intraperitoneal injection) prevents the decrease in SIR2 expression and the increases in the p38MAPK and p53 levels and the dephosphorylation of histone H3 in the kidney of STZ-induced diabetic rats, suggesting that SIR2 is involved in the beneficial effects of resveratrol in the kidneys [38]. Furthermore, Wu et al. demonstrated that resveratrol has protective effects on diabetic kidneys by modulating the SIRT1/FOXO1 pathway [39]. They demonstrated that FOXO1 activity is reduced, with a concomitant decrease in the expression of catalase, a FOXO1 target gene, and that SIRT1 expression decreased in the renal cortex of STZ-induced diabetic rats, resulting in enhanced renal oxidative stress. Treatment with resveratrol increased the renal FOXO1 activity, catalase expression, and SIRT1 expression, leading to a reduction in oxidative stress.Moreover, in cultured mesangialcells, Xu et al. demonstrated that resveratrol exerts protective effects onhigh glucose-induced mitochondrial oxidative stress and mitochondrial dysfunction[40]. All of these protective effects of resveratrol were blocked by the knockdown of SIRT1 and by EX-527, a specific inhibitor of SIRT1.

Chen et al. reported that resveratrol treatment improved diabetes-induced glomerular hypertrophy and urinary albumin excretion;reduced the expression ofglomerular fibronectin, collagen IV, and transforming growth factor (TGF)-*β*; reduced the thickness of the glomerular basement membrane; and reduced nephrin expression in the kidneys of STZ-induced diabetic rats, possibly through the inhibition of the phosphorylation of Smad2, Smad3, and ERK1/2 [41]. However, the mechanism by which resveratrol inhibits Smad2, Smad3, and ERK1/2 phosphorylation remains unknown.

### 3.2. Drug-Induced Renal Injury Model

Cisplatin is a chemotherapeutic agent that is widely used to treat malignant tumors. As the most common adverse effect of cisplatin, nephrotoxicity is an important dose-limiting factor in cisplatin treatment. The nephrotoxicity of cisplatin is induced directly by DNA damage, inflammation, and oxidative stress in the proximal tubules ofthe S3 segment in the outer medulla and the corticomedullary region of the kidney[42].Amaral et al. reported that pretreatment with resveratrol(25 mg/kg, intraperitoneal injection) attenuatedsigns of cisplatin-induced renal injury, such as tubular cell apoptosis and inflammation and renal dysfunction, by reducing the level of oxidative stress and inhibiting inflammation[43]. In addition, Kim et al. showed that SIRT1 activation by resveratrol reducesthe cisplatin-induced acetylation of p53, apoptosis, and cytotoxicity in cultured mouse proximal tubular cells [44]. SIRT1 expression and activity after 3 days of cisplatin treatment have been shown to decrease in the kidneys; however, the administration of resveratrol amelioratedthe decreases of SIRT1 activation and the glomerular filtration rate and the increases of tubular cell apoptosis and urinary Kim-1 excretion, which is induced by cisplatin.

Other studieshave shown that resveratrol attenuated renal injurycaused by several drugs, including glycerol[45, 46], gentamicin[47, 48], and cyclosporine[49], by reducing oxidative stress, as one of the mechanisms of the renal protective effect of resveratrol.

### 3.3. Aldosterone-Induced Kidney Injury

Aldosterone and its activation pathway through mineralocorticoid receptor contribute to podocytes injuries and progression of proteinuric kidney disease. Yuan et al. reported that SIRT1/PGC-1*α* axis in mitochondria ameliorated aldosterone-induced podocytes injuries[50]. They found that aldosterone suppressed SIRT1 and PGC-1*α* activation in cultured podocytes, resulting in increased podocytes apoptosis and the loss of slit diaphragm proteins, including nephrin and podocin, accompanied with mitochondrial dysfunction. SIRT1 activation protected against aldosterone-induced podocytes injuries with mitochondrial dysfunction, by inhibiting both apoptosis and loss of slit diaphragm proteins, through deacetylation and activation of PGC-1*α*. Treatment with resveratrol prevented aldosterone-induced podocytes apoptosis and mitochondrial dysfunction and restored expression of nephrin and podocin in vitro and vivo model, through activation of the SIRT1/PGC-1*α* axis.

### 3.4. Ischemia-Reperfusion and Sepsis-Induced Kidney Injuries

Renal ischemia is a commoncourse of AKI. Reperfusion is essential for the survival of ischemic renal tissue; however, reperfusion also contributes to additional renal damages[51]. Oxidative stress plays a crucial role in ischemia-reperfusion injury of the kidney. Several studieshave demonstrated that resveratrol exerts protective effects against ischemia-reperfusion injury in the kidneys, as well as the heart and brain injury, by reducing oxidative stress and several other mechanisms.Giovannini et al. reported that the pretreatment of rats with resveratrol (0.23 *μ*g/kg) reduced the mortality rate of ischemic rats from 50% to 10% and reduced the extent of renal damage, as reflected by glomerular dysfunction, tubular cell necrosis, inflammatory cell infiltration, glomerular thrombosis, urinary IL-6 excretion, and oxidative stress[52]. Peroxynitrite (ONOO^−^), which is generated by the reaction of NO with superoxide, is a powerful oxidizing RNS and causes protein nitration, DNA damage, and mitochondrial dysfunction, leading to endothelial and epithelial dysfunction.Treatment with NG-nitro-L-arginine methyl ester (L-NAME), which is a nitric oxide synthase inhibitor, abolished the effects of resveratrol on ischemic kidneys, suggesting that resveratrol protects the kidneys from ischemia-reperfusion injury through a nitric oxide-dependent mechanism. Chander and Chopra also showed that pretreatment with resveratrol (5 mg/kg) attenuates renal ischemia-reperfusion injury through NO release in rats [53].Resveratrol may enhance the enzymatic activity of endothelial NOS (eNOS) through phosphorylation by AMPK [54]or deacetylation by SIRT1 [55], possibly leading to the production of NO and protectingvascular tissues, including the kidneys. The transcriptional activity of eNOS is also increased by resveratrol-induced FOXO activation via SIRT1[56].

The development of AKIis a common complication during severe sepsis and more than doubles the mortality rate to nearly 75%[57]. When severe sepsis develops, the dysfunction of the renal microcirculation, which is induced by increased oxidative stress, especially as the result of reactive nitrogen species (RNS), contributes to the progression of AKI[58–60]. Holthoff et al. investigated the effects of resveratrol on sepsis-induced AKI using the cecal ligation and puncture (CLP) murine model[61]. Resveratrol restoredthe renal microcirculation and scavenged reactive nitrogen species, thus protecting the tubular cells in the kidney during sepsis. Furthermore, the administration of resveratrol to septic mice at 6, 12, and 18 hr resulted in a significant improvement in survival compared with that of the vehicle-treated mice.

### 3.5. Obstructed Kidneys

Renal fibrosis is the hallmark of progressive renal disease and is recognized as the final common pathway of glomerular sclerosis and tubule-interstitial fibrosis. The unilateral ureteral obstruction (UUO) model is widely used to investigate the mechanisms of renal fibrosis[62]. The TGF-*β*/Smad3 signaling pathway plays a central role in the pathogenesis of renal fibrosis. Li et al. reported that resveratrol reversed the acetylation of Smad3 and inhibited the TGF-*β*-induced upregulation of fibrosis-related genes, such as collagen IV and fibronectin, through SIRT1 activation in the interstitial lesion of the obstructed kidney[63].

### 3.6. Aging Kidney

Aging causes progressive postmaturational deterioration of tissues and organs, leading to impaired tissue function, increased vulnerability to stress, and death. Kidney is one of the typical target organs of age-associated tissue damage, and the high incidence of CKD in the elderly is a health problem worldwide.

Kume et al. found that mitochondrial damage in aged kidneys is associated with a decrease in SIRT1 activation [64]. In the renal proximal tubular cells of aged mice, autophagy in response to renal hypoxia is decreased, resulting in renal dysfunction and histological renal fibrosis. CR-mediated renal SIRT1 activation deacetylates and activates FOXO3a transcriptional activity, leading to the recovery of Bnip3-mediated autophagy, even in aged kidneys. These findings indicate that SIRT1 is a crucial target in aging kidneys; therefore, resveratrol is expected to prevent renal aging.

## 4. Conclusions

**Figure 2:**
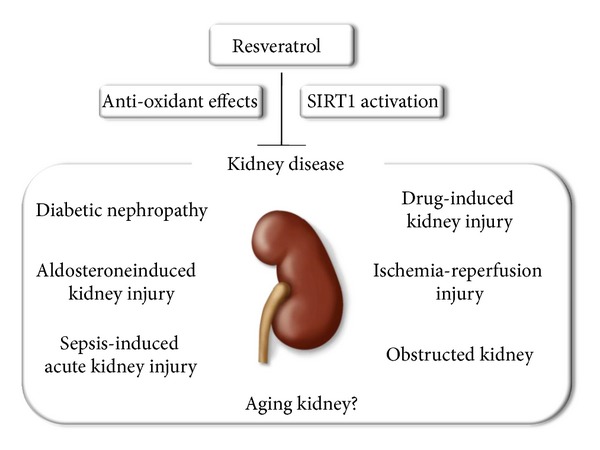
Resveratrol can prevent renal injury, including diabetic nephropathy, drug-induced renal injury, aldosterone-induced renal injury, ischemia-reperfusion injury, sepsis-induced kidney injury, and obstructed kidney, through its antioxidant effects and SIRT1 activation.

Resveratrol can exert protective effects against both acute and chronic kidney injuries through its antioxidanteffects and ability to activate SIRT1 (Figure 2). Therefore, resveratrol should be a useful additional treatment for preventing renal injury. However, it remains unclear whether resveratrol has beneficial effects on kidney diseases in humans and other animal models of renal diseases. In addition, a number of recent studies indicate that many of the protective effects of resveratrol could be mediated by SIRT1-independent mechanisms. Among them, the activation of mammalian target of rapamycin (mTOR) signaling pathway is involved in the pathogenesis for several kidney diseases, such as diabetic nephropathy [65–67] and the autosomal dominant polycystic kidney disease [68]. Liu et al. reported that RSV increases the association between mTOR and the DEP-domain-containing and mTOR-interactive protein (DEPTOR), an identified negative regulator of mTOR [69]. Therefore, resveratrol is expected to protect the kidney by the inhibition of mTOR pathway. Further studies are necessary to verify the beneficial effects of this compound in humans and other animals of kidney diseases and to clarify the detailed mechanism for the renal protective effect of resveratrol.

## References

[1] Levin A, Stevens PE (2013). Summary of KDIGO guideline: behind the scenes, need for guidance, and a framework for moving forward. *Kidney International*.

[2] Uchino S, Kellum JA, Bellomo R (2005). Acute renal failure in critically ill patients: a multinational, multicenter study. *Journal of the American Medical Association*.

[3] Chawla LS, Kimmel PL (2012). Acute kidney injury and chronic kidney disease: an integrated clinical syndrome. *Kidney International*.

[4] Bertelli AAA, Das DK (2009). Grapes, wines, resveratrol, and heart health. *Journal of Cardiovascular Pharmacology*.

[5] Renaud S, de Lorgeril M (1992). Wine, alcohol, platelets, and the French paradox for coronary heart disease. *The Lancet*.

[6] Catalgol B, Batirel S, Taga Y, Ozer NK (2012). Resveratrol: French paradox revisited. *Frontiers in pharmacology*.

[7] Fontana L, Partridge L, Longo VD (2010). Extending healthy life span-from yeast to humans. *Science*.

[8] Cohen HY, Miller C, Bitterman KJ (2004). Calorie restriction promotes mammalian cell survival by inducing the SIRT1 deacetylase. *Science*.

[9] Guarente L (2011). Sirtuins, aging, and medicine. *The New England Journal of Medicine*.

[10] Kitada M, Kume S, Takeda-Watanabe A, Kanasaki K, Koya D (2013). Sirtuins and renal diseases: relationship with aging and diabetic nephropathy. *Clinical Science*.

[11] Howitz KT, Bitterman KJ, Cohen HY (2003). Small molecule activators of sirtuins extend Saccharomyces cerevisiae lifespan. *Nature*.

[12] Kitada M, Kume S, Kanasaki K (2013). Sirtuins as possible drug targets in type 2 diabetes. *Current Drug Targets*.

[13] Dröge W (2002). Free radicals in the physiological control of cell function. *Physiological Reviews*.

[14] Droge W, Roach CR, Wagner DP, Hackett HP (2003). Oxidative stress and aging. *Hypoxia*.

[15] Holthoff JH, Woodling KA, Doerge DR, Burns ST, Hinson JA, Mayeux PR (2010). Resveratrol, a dietary polyphenolic phytoalexin, is a functional scavenger of peroxynitrite. *Biochemical Pharmacology*.

[16] Leonard SS, Xia C, Jiang B-H (2003). Resveratrol scavenges reactive oxygen species and effects radical-induced cellular responses. *Biochemical and Biophysical Research Communications*.

[17] Pervaiz S, Holme AL (2009). Resveratrol: its biologic targets and functional activity. *Antioxidants and Redox Signaling*.

[18] Robb EL, Winkelmolen L, Visanji N, Brotchie J, Stuart JA (2008). Dietary resveratrol administration increases MnSOD expression and activity in mouse brain. *Biochemical and Biophysical Research Communications*.

[19] Mokni M, Elkahoui S, Limam F, Amri M, Aouani E (2007). Effect of resveratrol on antioxidant enzyme activities in the brain of healthy rat. *Neurochemical Research*.

[20] Kitada M, Kume S, Imaizumi N, Koya D (2011). Resveratrol improves oxidative stress and protects against diabetic nephropathy through normalization of Mn-SOD dysfunction in AMPK/SIRT1- independent pathway. *Diabetes*.

[21] Colman RJ, Anderson RM, Johnson SC (2009). Caloric restriction delays disease onset and mortality in rhesus monkeys. *Science*.

[22] Fontana L, Meyer TE, Klein S, Holloszy JO (2004). Long-term calorie restriction is highly effective in reducing the risk for atherosclerosis in humans. *Proceedings of the National Academy of Sciences of the United States of America*.

[23] Meyer TE, Kovács SJ, Ehsani AA, Klein S, Holloszy JO, Fontana L (2006). Long-term caloric restriction ameliorates the decline in diastolic function in humans. *Journal of the American College of Cardiology*.

[24] Imai S-I, Armstrong CM, Kaeberlein M, Guarente L (2000). Transcriptional silencing and longevity protein Sir2 is an NAD-dependent histone deacetylase. *Nature*.

[25] Bordone L, Cohen D, Robinson A (2007). SIRT1 transgenic mice show phenotypes resembling calorie restriction. *Aging Cell*.

[26] Bellot G, Garcia-Medina R, Gounon P (2009). Hypoxia-induced autophagy is mediated through hypoxia-inducible factor induction of BNIP3 and BNIP3L via their BH3 domains. *Molecular and Cellular Biology*.

[27] Steinberg GR, Kemp BE (2009). AMPK in health and disease. *Physiological Reviews*.

[28] Park S-J, Ahmad F, Philp A (2012). Resveratrol ameliorates aging-related metabolic phenotypes by inhibiting cAMP phosphodiesterases. *Cell*.

[29] Price NL, Gomes AP, Ling AJY (2012). SIRT1 is required for AMPK activation and the beneficial effects of resveratrol on mitochondrial function. *Cell Metabolism*.

[30] Hubbard BP, Gomes AP, Dai H (2013). Evidence for a common mechanism of SIRT1 regulation by allosteric activators. *Science*.

[31] Kitada M, Zhang Z, Mima A, King GL (2010). Molecular mechanisms of diabetic vascular complications. *Journal of Diabetes Investigation*.

[32] Sharma S, Anjaneyulu M, Kulkarni SK, Chopra K (2006). Resveratrol, a polyphenolic phytoalexin, attenuates diabetic nephropathy in rats. *Pharmacology*.

[33] Palsamy P, Subramanian S (2011). Resveratrol protects diabetic kidney by attenuating hyperglycemia-mediated oxidative stress and renal inflammatory cytokines via Nrf2-Keap1 signaling. *Biochimica et Biophysica Acta*.

[34] Kim MY, Lim JH, Youn HH (2013). Resveratrol prevents renal lipotoxicity and inhibits mesangial cell glucotoxicity in a manner dependent on the AMPK-SIRT1-PGC1*α* axis in db/db mice. *Diabetologia*.

[35] Zhang L, Pang S, Deng B (2012). High glucose induces renal mesangial cell proliferation and fibronectin expression through JNK/NF-*κ*B/NADPH oxidase/ROS pathway, which is inhibited by resveratrol. *International Journal of Biochemistry and Cell Biology*.

[36] Ding D-F, You N, Wu X-M (2010). Resveratrol attenuates renal hypertrophy in early-stage diabetes by activating AMPK. *American Journal of Nephrology*.

[37] Lee M-J, Feliers D, Sataranatarajan K (2010). Resveratrol ameliorates high glucose-induced protein synthesis in glomerular epithelial cells. *Cellular Signalling*.

[38] Tikoo K, Singh K, Kabra D, Sharma V, Gaikwad A (2008). Change in histone H3 phosphorylation, MAP kinase p38, SIR 2 and p53 expression by resveratrol in preventing streptozotocin induced type I diabetic nephropathy. *Free Radical Research*.

[39] Wu L, Zhang Y, Ma X, Zhang N, Qin G (2012). The effect of resveratrol on FoxO1 expression in kidneys of diabetic nephropathy rats. *Molecular Biology Reports *.

[40] Xu Y, Nie L, Yin Y-G (2012). Resveratrol protects against hyperglycemia-induced oxidative damage to mitochondria by activating SIRT1 in rat mesangial cells. *Toxicology and Applied Pharmacology*.

[41] Chen K-H, Hung C-C, Hsu H-H, Jing Y-H, Yang C-W, Chen J-K (2011). Resveratrol ameliorates early diabetic nephropathy associated with suppression of augmented TGF-*β*/smad and ERK1/2 signaling in streptozotocin-induced diabetic rats. *Chemico-Biological Interactions*.

[42] Miller RP, Tadagavadi RK, Ramesh G, Reeves WB (2010). Mechanisms of cisplatin nephrotoxicity. *Toxins*.

[43] Do Amaral CL, Francescato HDC, Coimbra TM (2008). Resveratrol attenuates cisplatin-induced nephrotoxicity in rats. *Archives of Toxicology*.

[44] Kim DH, Jung YJ, Lee JE (2011). Sirt1 activation by resveratrol ameliorates cisplatin-induced renal injury through deacetylation of p53. *American Journal of Physiology: Renal Physiology*.

[45] de Jesus Soares T, Volpini RA, Francescato HDC, Costa RS, da Silva CGA, Coimbra TM (2007). Effects of resveratrol on glycerol-induced renal injury. *Life Sciences*.

[46] Chander V, Chopra K (2006). Protective effect of resveratrol, a polyphenolic phytoalexin on glycerol-induced acute renal failure in rat kidney. *Renal Failure*.

[47] Morales AI, Buitrago JM, Santiago JM, Fernández-Tagarro M, López-Novoa JM, Pérez-Barriocanal F (2002). Protective effect of trans-Resveratrol on gentamicin-induced nephrotoxicity. *Antioxidants and Redox Signaling*.

[48] Silan C, Uzun Ö, Çomunoğlu NÜ, Gokçen S, Bedirhan S, Cengiz M (2007). Gentamicin-induced nephrotoxicity in rats ameliorated and healing effects of resveratrol. *Biological and Pharmaceutical Bulletin*.

[49] Chander V, Tirkey N, Chopra K (2005). Resveratrol, a polyphenolic phytoalexin protects against cyclosporine-induced nephrotoxicity through nitric oxide dependent mechanism. *Toxicology*.

[50] Yuan Y, Huang S, Wang W (2012). Activation of peroxisome proliferator-activated receptor-gamma coactivator 1alpha ameliorates mitochondrial dysfunction and protects podocytes from aldosterone-induced injury. *Kidney International*.

[51] Weight SC, Bell PRF, Nicholson ML (1996). Renal ischaemia-reperfusion injury. *British Journal of Surgery*.

[52] Giovannini L, Migliori M, Longoni BM (2001). Resveratrol, a polyphenol found in wine, reduces ischemia reperfusion injury in rat kidneys. *Journal of Cardiovascular Pharmacology*.

[53] Chander V, Chopra K (2006). Protective effect of nitric oxide pathway in resveratrol renal ischemia-reperfusion injury in rats. *Archives of Medical Research*.

[54] Xu Q, Hao X, Yang Q, Si L (2009). Resveratrol prevents hyperglycemia-induced endothelial dysfunction via activation of adenosine monophosphate-activated protein kinase. *Biochemical and Biophysical Research Communications*.

[55] Arunachalam G, Yao H, Sundar IK, Caito S, Rahman I (2010). SIRT1 regulates oxidant- and cigarette smoke-induced eNOS acetylation in endothelial cells: role of resveratrol. *Biochemical and Biophysical Research Communications*.

[56] Xia N, Strand S, Schlufter F (2013). Role of SIRT1 and FOXO factors in eNOS transcriptional activation by resveratrol. *Nitric Oxide*.

[57] Heemskerk S, Masereeuw R, Russel FGM, Pickkers P (2009). Selective iNOS inhibition for the treatment of sepsis-induced acute kidney injury. *Nature Reviews Nephrology*.

[58] Mayeur N, Minville V, Jaafar A (2011). Morphologic and functional renal impact of acute kidney injury after prolonged hemorrhagic shock in mice. *Critical Care Medicine*.

[59] Wu L, Gokden N, Mayeux PR (2007). Evidence for the role of reactive nitrogen species in polymicrobial sepsis-induced renal peritubular capillary dysfunction and tubular injury. *Journal of the American Society of Nephrology*.

[60] Wu L, Tiwari MM, Messer KJ (2007). Peritubular capillary dysfunction and renal tubular epithelial cell stress following lipopolysaccharide administration in mice. *American Journal of Physiology: Renal Physiology*.

[61] Holthoff JH, Wang Z, Seely KA, Gokden N, Mayeux PR (2012). Resveratrol improves renal microcirculation, protects the tubular epithelium, and prolongs survival in a mouse model of sepsis-induced acute kidney injury. *Kidney International*.

[62] Chevalier RL, Forbes MS, Thornhill BA (2009). Ureteral obstruction as a model of renal interstitial fibrosis and obstructive nephropathy. *Kidney International*.

[63] Li J, Qu X, Ricardo SD, Bertram JF, Nikolic-Paterson DJ (2010). Resveratrol inhibits renal fibrosis in the obstructed kidney: potential role in deacetylation of Smad3. *American Journal of Pathology*.

[64] Kume S, Uzu T, Horiike K (2010). Calorie restriction enhances cell adaptation to hypoxia through Sirt1-dependent mitochondrial autophagy in mouse aged kidney. *Journal of Clinical Investigation*.

[65] Inoki K, Mori H, Wang J (2011). mTORC1 activation in podocytes is a critical step in the development of diabetic nephropathy in mice. *Journal of Clinical Investigation*.

[66] Gödel M, Hartleben B, Herbach N (2011). Role of mTOR in podocyte function and diabetic nephropathy in humans and mice. *Journal of Clinical Investigation*.

[67] Sakaguchi M, Isono M, Isshiki K, Sugimoto T, Koya D, Kashiwagi A (2006). Inhibition of mTOR signaling with rapamycin attenuates renal hypertrophy in the early diabetic mice. *Biochemical and Biophysical Research Communications*.

[68] Lieberthal W, Levine JS (2012). Mammalian target of rapamycin and the kidney. II. Pathophysiology and therapeutic implications. *American Journal of Physiology: Renal Physiology*.

[69] Liu M, Wilk SA, Wang A (2010). Resveratrol inhibits mTOR signaling by promoting the interaction between mTOR and DEPTOR. *Journal of Biological Chemistry*.

